# 2013: an year of celebration and joy for all of us

**DOI:** 10.5935/1808-8694.20130001

**Published:** 2015-10-14

**Authors:** Wilma Anselmo-Lima, José Eduardo Lutaif Dolci

**Affiliations:** Associate Professor - Otorhinolaryngology Division, Department of Ophthalmology and Otorhinolaryngology and Head and Neck Surgery - School of Medicine of Ribeirão Preto; Full Professor and Director of the School of Medicine of Santa Casa de São Paulo

The Brazilian Journal of Otorhinolaryngology (BJORL) celebrates 80 years (1933-2013) of science and progress; this means 80 years of contribution to the advancement of our specialty.

We want to pay a very special tribute to all editors who have made our journal an entity truly representative of the scientific community of otorhi-nolaryngologists.

We would also like to highlight the progress made in the year 2012: we hired a CEO who has provided an important support to the editorial production; shown the papers “ahead of print” at the website; and a DOI number assignment to each paper published. Additionally, our members may already have access to issues in mobile devices such as I-phones and I-pads. This is technology combined with science!

Finally, we want to thank our colleagues who participated with exemplary dedication as associate editors and reviewers in this biennium, 2011 and 2012, a period in which we won our indexing to ISI, and we wish to extend a warm welcome to new members.

Our future as a high level journal depends on each of us; thus, we count on the support from all of you, so as to increasingly have quality publications in our BJORL.

A great 2013 to all!

